# COMPARISON OF MOTION SENSOR AND HEART RATE MONITOR FOR ASSESSMENT OF PHYSICAL ACTIVITY INTENSITY IN STROKE OUTPATIENT REHABILITATION SESSIONS: AN OBSERVATIONAL STUDY

**DOI:** 10.2340/jrm.v56.40559

**Published:** 2024-06-25

**Authors:** Stéphanie GONCALVES, Stéphane MANDIGOUT, Morgane LE BOURVELLEC, Noémie C. DUCLOS

**Affiliations:** 1 University Limoges, HAVAE, UR 20217, Limoges; 2 MOVE UR 20296, Poitiers University; 3 University Bordeaux, INSERM, BPH, U1219, Bordeaux, France

**Keywords:** accelerometery, intensity, physical activity, rehabilitation, sensor, stroke

## Abstract

**Objective:**

To compare the estimation of time spent on 4 categories of physical activity intensity (sedentary behaviour, light physical activity, moderate physical activity, and vigorous physical activity) between a motion sensor and a heart rate monitor during a stroke outpatient rehabilitation session.

**Design:**

A multicentre cross-sectional observational study.

**Subjects/Patients:**

Participants with stroke (> 6 months) undergoing outpatient rehabilitation sessions.

**Methods:**

Participants wore the SenseWear Armband motion sensor and the Polar H10 heart rate monitor during 2 rehabilitation sessions. The times estimated by each device were compared using a generalized linear mixed model and post-hoc tests.

**Results:**

Ninety-nine participants from 29 clinics were recruited and data from 146 sessions were included in the analysis. The estimated times depended on the devices and the physical activity intensity category (F = 135, *p* < 0.05). The motion sensor estimated more time spent in sedentary behaviour and less time spent in moderate physical activity and vigorous physical activity than the heart rate monitor.

**Conclusion:**

The motion sensor and heart rate monitor provide different estimates of physical activity intensity during stroke rehabilitation. Further research is needed to establish the most appropriate device for each physical activity category.

LAY ABSTRACT

Physical activity is important after a stroke to maintain physical capacity and prevent stroke recurrence. Our study compared 2 devices commonly used to track physical activity level during stroke rehabilitation sessions: a motion sensor and a heart rate monitor. We wanted to see how well these devices estimated time spent on performing different levels of physical activity, i.e., sedentary behaviour, light physical activity, moderate physical activity, and vigorous physical activity. We found that the motion sensor and heart rate monitor did not always agree on how much time was spent on each activity level. Specifically, when measured with the motion sensor, the individuals appeared to be more sedentary and engaged in fewer minutes of moderate and vigorous activities than when measured by a heart rate monitor. Further research should investigate which device is more accurate for monitoring physical activity in this context, to help clinicians optimize rehabilitation programmes for people after stroke.

To improve health, the WHO 2020 guidelines on physical activity for the general population recommend allocating time to each category of physical activity intensity (PAI), i.e., light (LPA), moderate (MPA), and vigorous physical activity (VPA) (1). They also include specific recommendations for individuals with disabilities who face a heightened risk of serious health consequences resulting from inactivity and sedentary behaviour (SB) compared with the general population (2). Exercise-based rehabilitation plays a key role in tackling physical inactivity and sedentary behaviour in these groups (3). Therefore, accurately assessing the time spent on each PAI category during rehabilitation sessions is crucial to evaluate adherence to the guidelines (4).

Stroke is a leading cause of long-term disability worldwide (5). Incorporating 20 min of aerobic exercise (i.e., MPA) within rehabilitation sessions is recommended to reduce the risk of subsequent stroke (6). Insufficient PAI during rehabilitation interventions may lead to lower health benefits. However, excessive PAI poses safety concerns, risking harmful overexertion (7) and potentially reducing patient adherence (8). Knowing that clinicians frequently overestimate the time spent on activity during rehabilitation sessions by 30% (9) and the lack of validity of individuals’ ratings of perceived exertion (10), quantifying PAI objectively during rehabilitation sessions is essential to guide rehabilitation.

PAI during neurorehabilitation interventions can be estimated objectively using either absolute or relative measures (11). Absolute measures provide insights into PAI related to movement (12). The most common devices used to measure absolute PAI are motion sensors, configured to convert data, notably acceleration, into energy expenditure or PAI categories (4). They are a feasible method to track PAI during inpatient stroke rehabilitation (13). However, absolute measures may misclassify PAI in deconditioned individuals, such as those after stroke (11). In contrast, relative measures of PAI consider the individual’s physical condition by focusing on physiological responses, such as maximal heart rate (HRmax) or maximal oxygen consumption (11). The percentage of HRmax is a commonly used PAI measure during exercise sessions post-stroke (14) and heart rate monitors are relevant devices to estimate relative PAI (4).

Many observational studies have used either a heart rate monitor (15, 16) or a motion sensor (17, 18) to document PAI during post-stroke rehabilitation. However, their results are conflicting, with an estimation of 10% of therapy spent in moderate to vigorous physical activity using a motion sensor (18) vs 45% with a heart rate monitor (16). Given the comparable settings in the different studies, it is reasonable to assume that these discrepancies resulted from differences between the devices. Nevertheless, no studies have directly compared PAI estimations between motion sensors and heart rate monitors within the same session.

The main objective of this study was to compare the estimated time spent on SB, LPA, MPA, and VPA, during stroke outpatient rehabilitation sessions between a motion sensor and a heart rate monitor.

## METHODS

### Study design and setting

This research was part of a larger study to evaluate PAI during routine outpatient rehabilitation sessions in individuals in the chronic phase of stroke. It was a multicentre cross-sectional observational study conducted between January 2021 and September 2022 in the Nouvelle-Aquitaine Region of France. Ethical approval was granted by the Committee for the Protection of Persons (CNRIPH 20.01.16.63227). All participants provided written informed consent before their involvement. The study was carried out in accordance with the Helsinki Declaration and is reported according to the STROBE guidelines (Table SI).

### Participants

Owing to the exploratory nature of this study, no formal sample size was determined. Eligible participants were recruited by contacting all outpatient physiotherapists within a 20 km radius of Limoges, Bordeaux, and Périgueux in France. Inclusion criteria were: (a) community-dwelling individuals in the chronic phase of stroke (> 6 months since onset) and (b) undergoing rehabilitation sessions. Exclusion criteria were (a) contraindications to physical activity, such as uncontrolled hypertension or acute musculo-skeletal conditions preventing movement, (b) severe cognitive impairment (Mini-Mental State Examination [MMSE] < 11 (19)), (c) hydrotherapy sessions (as the motion sensor could not be used in water).

### Procedure

Each volunteer arrived 15 min before the rehabilitation session to confirm eligibility, complete questionnaires and clinical tests to characterize the sample, and set up the equipment. Rehabilitation sessions typically lasted 40 min and were held twice per week. These sessions included a variety of exercises such as endurance, balance, and functional lower limb training, tailored to the individual’s abilities and rehabilitation goals. Two sessions per participant were recorded to cover a wide range of activities.

### Devices

Participants were simultaneously equipped with a multisensory activity tracker and a heart rate monitor. They then engaged in their rehabilitation session as usual.

The SenseWear Armband (SWA) (Body Media, Pittsburgh, PA, USA) multisensory tracker was placed over the triceps muscle of the non-paretic arm, as recommended in people with stroke (20). The SWA includes a bi-axial accelerometer and sensors that measure galvanic skin response, skin temperature, and heat flux. Data were computed using the proprietary algorithm for each minute of device wear. Among commercially available wearable sensors, the SWA stands out for its precision in estimating energy expenditure and PAI in daily tasks in people after stroke (21).

The Polar H10 heart rate monitor (Polar Electro Oy, Kemple, Finland) was placed around the patient’s chest. Heart rate data were recorded at 1-sec intervals and were sent wirelessly via Bluetooth to a smartphone, using the Polar Beat app. The Polar H10 has been validated against electrocardiography during exercise (22).

### Outcomes

The primary outcome was the estimated time spent on each PAI category (SB, LPA, MPA, VPA) during 2 rehabilitation sessions, measured by each device.

We collected self-reported demographic data and details regarding stroke, including age, sex, time since stroke, side of stroke, use of beta-blocking medication, and body mass index (BMI). Motor function was measured using the Motricity Index, which scores from 0 to 100 (23). Cognitive impairment was evaluated with the Mini-Mental State Examination (MMSE), a 30-point questionnaire with cut-off scores (≥ 27 indicates no impairment, 21–26 indicates mild impairment, and ≤ 10 severe impairment) (19). Comfortable gait speed was measured by the 10-Meter Walk Test (24). This test involved timing the walk over the middle 10-meter section of a 14-meter track, with the average time from 2 trials recorded.

### Data processing

After each session, SWA data were downloaded and processed using the algorithms developed by the manufacturer (SenseWear Professional software, version 8.1; https://bodymedia-sensewear.software.informer.com/). PAI estimation from this device was based on energy expenditure in Metabolic Equivalent of Task (MET). Raw heart rate data were downloaded from the Polar Flow website (Polar Electro Oy; https://www.polar.com/fi). PAI estimation from this device was expressed as a percentage of age-predicted maximal heart rate (%HRmax). In accordance with recent guidelines (25), maximal heart rate was predicted using the Gellish formula: 206.9–0.67 x age (26), or the Brawner formula for participants taking beta-blockers: 164–0.7 x age (27).

The data were excluded from the analysis when (1) the cumulative signal loss exceeded 10% of the overall session time recorded in minutes using a stopwatch, and (2) the recording duration during the session differed by more than 10% between the devices.

PAI thresholds for both devices were based on the French National Health Authority guidelines (28): time spent in SB: 0.9 < MET < 1.5 or % HRmax < 40%; LPA: 1.6 < MET < 2.9 or 40% < % HRmax < 55%; MPA: 3 < MET < 5.9 or 55% < % HRmax < 70%; and VPA: MET ≥ 6 or % HRmax ≥70%. Time spent on each PAI category was determined using a custom-made MATLAB 2019b (The MathWorks; https://uk.mathworks.com/) script.

### Statistical analysis

Statistical analysis was carried out using R studio version 4.2.1 (R Foundation for Statistical Computing, Vienna, Austria). The normality of distribution of continuous variables was tested by 1-sample Kolmogorov–Smirnov test. Continuous variables with normal distribution were presented as mean (standard deviation [SD]) and non-normal variables were reported as median (interquartile range [IQR]). For all analyses, a *p*-value less than 0.05 was considered statistically significant.

A model-based approach was adopted to comprehensively understand and compare measurement devices within and across different PAI categories. Because of violations of key assumptions of linear models, including heteroscedasticity, a generalized linear mixed model (GLMM) was employed, specifying a gamma distribution, suitable for time data and accounting for pseudoreplication of individuals in the dataset (2 sessions for each participant). The model was implemented using the *lme4* package in R. Interaction between the device and the PAI category was tested to assess the influence of the device on the estimated time spent in each PAI category. Post hoc analyses were conducted to explore further any differences using the *emmeans* package. This approach allowed for pairwise comparisons adjusting for multiple testing using the Tuckey procedure. Finally, additional boxplots were generated to analyse individual differences.

## RESULTS

Ninety-nine participants were recruited from 29 private outpatient clinics, and 198 sessions were recorded. Technical issues occurred in 18 sessions when a tablet with the Polar Team application was used but the raw heart rate data proved inaccessible. Polar H10 cumulative signal loss exceeding 10% of the total session time occurred in 28 sessions, and total recording durations differed by over 10% between devices in 5 sessions. The recording of the second session for 1 participant was not possible because of an allergy to a component of the SWA experienced during the first session. Thus, data from 146 sessions and 82 participants were included in the analysis (Fig. S1). Median (Q1, Q3) age of the participants was 68 (55, 73) years. Median time since stroke was 6.7 (2.6, 11.3) years. Median Motricity Index scores were 65 (44, 76)% for the lower limb and 58 (29, 84)% for the upper limb. Two participants were non-ambulatory. Participant characteristics are detailed in Table I.

**Table I:** Characteristics of participants

Sample characteristics (*n* = 82)	Total
Age (years), median (Q1, Q3)	68 (55, 73)
Sex, *n* males (%)	44 (53.7%)
Time since stroke (years), median (Q1, Q3)	6.7 (2.6, 11.3)
Side of stroke, *n* left (%)	34 (41.5)
Body mass index (kg/m^2^), mean (SD)	26.8 (4.5)
Beta-blocking medication, *n* (%)	17 (20.7%)
MMSE, median (Q1, Q3)	26 (22, 29)
10 MWT (m/s), median (Q1 ,Q3)	0.6 (0.3, 0.8)
Motricity Index LL (%), median (Q1, Q3)(Min–Max)	65 (44, 76) (1–100)
Motricity Index UL (%), median (Q1, Q3)(Min–Max)	58 (29, 84) (1–100)

SD: standard deviation; Q1, Q3: interquartile range; MMSE: Mini-Mental State Examination; 10MWT: 10-m walk test; LL: lower limb; UL: upper limb.

Median PAI estimation using the SWA was 21 (11, 34) min in SB, 11 (1, 21) min in LPA, 0 (0,6) min in MPA, and 0 (0,0) min in VPA. This corresponds to approximately 47 (23, 73)% of the total session duration spent in SB, 24 (2, 45)% in LPA, 0 (0, 13)% in MPA, and 0 (0, 0)% in VPA. Median PAI estimation using the Polar H10 was 0 (0, 0) min in SB, 9 (0, 21) min in LPA, 15 (2, 30) min in MPA, and 0 (0, 10) min in VPA. This corresponds to approximately 0 (0, 0)% of the total session duration spent in SB, 22.5 (0, 47)% in LPA, 37.5 (4, 67)% in MPA, and 0 (0, 22)% in VPA.

**Fig. 1:**
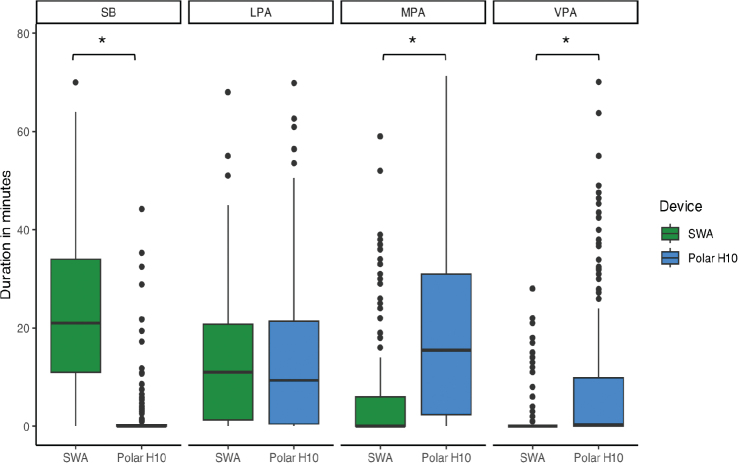
Time spent on each intensity category across sessions for both devices. SB: sedentary behaviour; LPA: light physical activity; MPA: moderate physical activity; VPA: vigorous physical activity; SWA: SenseWear armband.

**Fig. 2:**
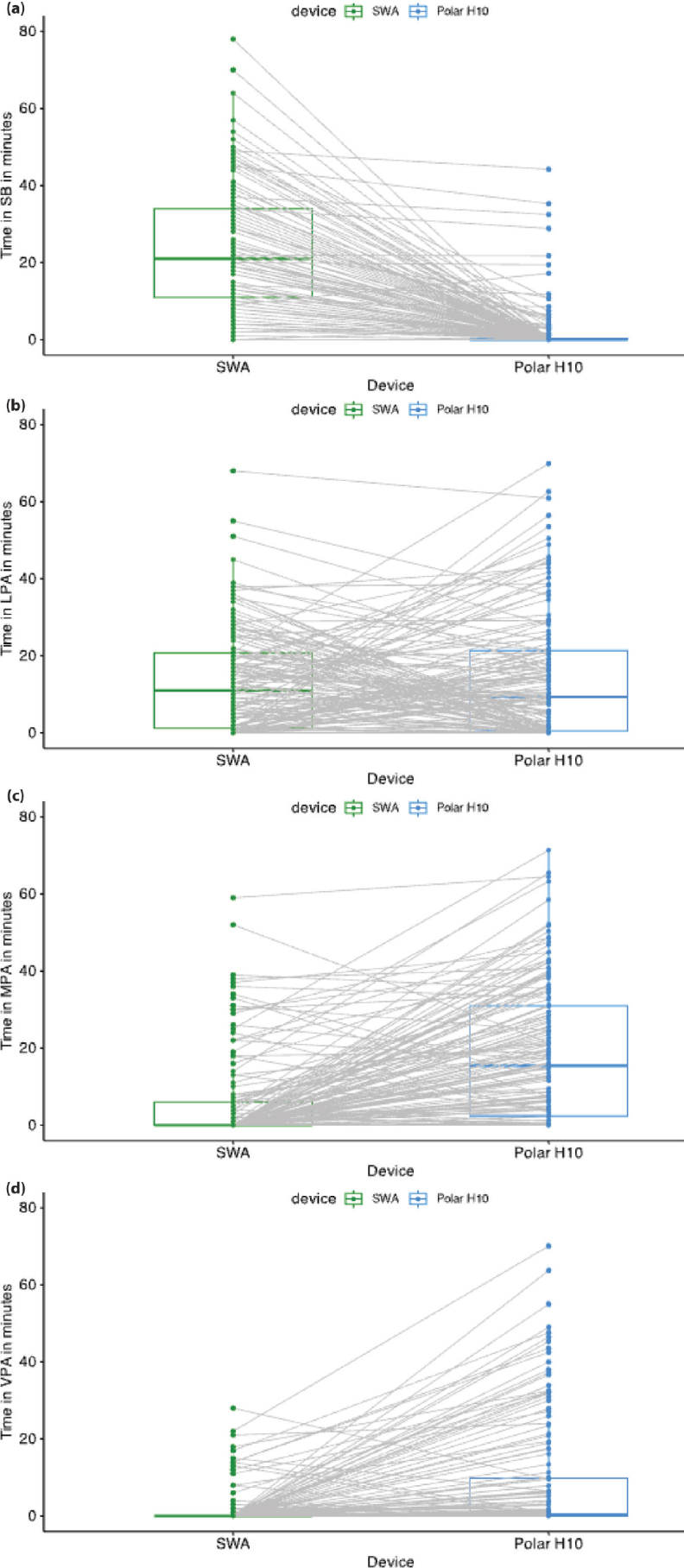
Estimations of physical activity intensity (PAI) for each session for each device: the grey lines connect the values from each device for a given session and a given individual. (a) Time spent on sedentary behaviour (SB). (b) Time spent on light physical activity (LPA). (c) Time spent on moderate physical activity (MPA). (d) Time spent on vigorous physical activity (VPA). SWA: SenseWear armand.

There was a significant interaction effect between the PAI category and device (F = 135, *p* < 0.05), meaning that the time estimated in each PAI category depended on the device used. Post-hoc tests (Table II) showed a significantly longer time spent on SB with SWA measurements than with the Polar H10 (z = –18.04, *p* < 0.001). In contrast, SWA measurements showed a significantly shorter time spent on MPA (z = 11.60, *p* < 0.001) and VPA (z = 11.30, *p* < 0.001) than the Polar H10. There was no significant difference between the SWA and Polar H10 for time spent in LPA (z = 1.91, *p* = 0.056) (Fig. 1). For each PAI category, individual variability was considerable (Fig. 2): each device provided different estimates of time spent on each PAI, sometimes higher or lower than the other device, depending on the individual.

**Table II:** Differences in time spent on each intensity category between Polar and SenseWear Armband (SWA)

Category	Contrast	Estimate	Standard Error	z-value	*p*-value
SB	Polar H10 - SWA	–2.264	0.127	–18.044	< 0.0001
LPA	Polar H10 - SWA	0.235	0.123	1.914	0.0557
MPA	Polar H10 - SWA	1.450	0.125	11.604	< 0.0001
VPA	Polar H10 - SWA	1.414	0.125	11.296	< 0.0001

Results are for the log (not the response) scale.

SB: sedentary behaviour; LPA: light physical activity; MPA: moderate physical activity; VPA: vigorous physical activity.

## DISCUSSION

This multicentre study found large differences in the estimated time spent on each PAI category between the SWA and the Polar H10. The differences varied across PAI categories, with large differences in the estimations of time spent on SB, MPA, and VPA between the devices. No statistical difference was found between the devices for LPA; however, Fig. 2 shows that, for some participants, the SWA estimation was higher, whereas for others, the Polar H10 estimation was higher.

The observed differences between the SWA and Polar H10 agreed with those of a recent systematic review that reported significant differences between absolute and relative measures of intensity during walking in healthy individuals (29).

The specific nature of the exercises performed during rehabilitation may have contributed to the differences found. SWA estimations of SB time were 21 min longer than Polar H10 estimations. Some exercises may not have been detected as active by the SWA (30) because of a lack of movement of the arm on which the SWA was positioned. For example, the SWA may not properly capture activities such as stationary cycling or isometric exercises (core exercises, stretching). Gait training using parallel bars or on a treadmill with minimal arm swing may also have been poorly detected. A study on the relationship between accelerometer and HR monitor measurements in older adults during exercise sessions found that an accelerometer placed on the hip failed to capture exercises involving balance and neuromotor function (31). This suggests that the position of the accelerometer should consider the specific exercises performed during rehabilitation sessions.

The differences could also be attributed to the limitations of the Polar H10 in estimating PAI. This device has low accuracy for tracking low-intensity activities (4). SB and LPA could be underestimated because of the high sensitivity of the heart rate to factors such as emotional state, hydration, and environmental conditions. Medication taken by the participants may also have influenced this measure. In total, 21% of participants took beta-blockers, which may have prevented their heart rates from reaching the rates considered to indicate moderate to vigorous intensity activity. However, the Polar H10 estimation of time spent on MPA was 14 min higher than that of the SWA. This difference could relate to the higher energy cost of activity for people with stroke than for healthy people (32). Indeed, an assessment of the intensity of activities of daily living in people after stroke using indirect calorimetry found that most activities induced MPA, whereas these activities were considered LPA for healthy individuals (33). Furthermore, the accelerometer thresholds used by manufacturers for each PAI category are calibrated for healthy individuals and they have been shown to underestimate PAI in individuals with chronic stroke (34).

Interestingly, the estimation of the time spent on LPA did not differ between devices. However, our additional plots (Fig. 2b) showed considerable intra-individual and inter-individual variability for this category, with one or the other device providing a higher or lower estimation of PAI depending on the individual. The elements discussed above (exercises, heart rate sensitivity, and energy cost) explain some of the same-session intra-individual variability between the devices. The inter-individual variability corroborates the results of Warner et al., who found variability in relative activity intensity for a given absolute intensity (29). This supports the importance of considering personalized approaches, based on individual characteristics and the session content.

From a clinical perspective, the differences in estimates between the motion sensor and the heart rate monitor suggest that clinicians should carefully consider device selection when interpreting PAI. The interindividual variability suggests that a one-size-fits-all approach is not appropriate. Crucially, the choice of the best-suited device should be driven by the intended purpose of the measurement, in combination with available resources. For instance, if the objective is to improve functional recovery through repetitive task training, the motion sensor is probably the most suitable device. Conversely, a heart rate monitor becomes the more obvious choice when aiming to enhance cardiorespiratory fitness through sessional aerobic exercise (25). Clinicians may need to use a combination of devices or additional observational assessments to gain a comprehensive understanding of a patient’s PAI.

The main limitation of this study is the lack of a gold standard, which prevents conclusions from being drawn as to the superiority of 1 device over the other. However, reference methods of physical activity assessment, such as indirect calorimetry, are challenging to implement in clinical practice. In addition, our issue with signal loss in over 13% of the sessions prevented the total data from being analysed. This underlines the well-acknowledged drawbacks of chest strap monitors. These drawbacks include potential challenges in setting up a Bluetooth connection with a paired smartphone, maintaining the monitor position during the exercise, reliance on humidity for conductivity and the discomfort caused by a tight strap necessary for proper functioning. Additionally, although the monitor’s positioning close to the heart ensures high accuracy and reliability (35), motion artefacts in the signal could be caused by trunk or upper limb movements (36). Moreover, we did not evaluate resting heart rate, which prevented us from employing the Karvonen formula. This equation is known for its increased accuracy compared with age-predicted maximal heart rate alone (37). Another limitation is the use of the Gellish formula, developed for non-disabled adults, which recently showed poor agreement with a cardiopulmonary exercise test in determining maximal heart rate (38). Despite using the adjusted Brawner formula for individuals taking beta-blockers, notable variability in the physiological response to exercise may persist (39). Variations in drug sensitivity among individuals could affect the PAI derived from this formula. Determining HRmax using a cardiopulmonary exercise test would more accurately estimate PAI (40). However, this test is rarely available in stroke outpatient rehabilitation settings. We aimed to assess PAI in a real-life situation to ensure the external validity of our results. This is the first study to compare PAI estimations between a motion sensor and a heart rate monitor in people after stroke in an outpatient setting. The external validity of our results was also strengthened by the large sample of participants, the wide range of levels of disability, and the large number of participating outpatient clinics.

In conclusion, this study showed large differences in the estimation of time spent on different PAI categories between a motion sensor and a heart rate monitor during rehabilitation sessions in people after stroke. These results highlight the need for careful interpretation of PAI estimations obtained from such devices, whether in clinical practice or research. Future research should compare these devices against gold standards to determine their respective accuracies and limitations in capturing physical activity intensity in the context of stroke rehabilitation.

## References

[1] Bull FC, Al-Ansari SS, Biddle S, Borodulin K, Buman MP, Cardon G, et al. World Health Organization 2020 guidelines on physical activity and sedentary behaviour. Br J Sports Med 2020; 54: 1451. DOI: 10.1136/bjsports-2020-102955 33239350 PMC7719906

[2] Martin Ginis KA, Van Der Ploeg HP, Foster C, Lai B, McBride CB, Ng K, et al. Participation of people living with disabilities in physical activity: a global perspective. Lancet 2021; 398: 443–455. DOI: 10.1016/S0140-6736(21)01164-8 34302764

[3] Limpens MM, Van Den Berg RJG, Den Uijl I, Sunamura M, Voortman T, Boersma E, et al. Physical activity and sedentary behaviour changes during and after cardiac rehabilitation: can patients be clustered? J Rehabil Med 2023; 55: jrm4343. DOI: 10.2340/jrm.v55.4343 37435716 PMC10364624

[4] Strath SJ, Kaminsky LA, Ainsworth BE, Ekelund U, Freedson PS, Gary RA, et al. Guide to the assessment of physical activity: clinical and research applications: a scientific statement from the American Heart Association. Circulation 2013; 128: 2259–2279. DOI: 10.1161/01.cir.0000435708.67487.da 24126387

[5] Feigin VL, Stark BA, Johnson CO, Roth GA, Bisignano C, Abady GG, et al. Global, regional, and national burden of stroke and its risk factors, 1990–2019: a systematic analysis for the Global Burden of Disease Study 2019. Lancet Neurol 2021; 20: 795–820. DOI: 10.1016/S1474-4422(21)00252-0 34487721 PMC8443449

[6] Billinger SA, Arena R, Bernhardt J, Eng JJ, Franklin BA, Johnson CM, et al. Physical activity and exercise recommendations for stroke survivors: a statement for healthcare professionals from the American Heart Association/American Stroke Association. Stroke 2014; 45: 2532–2553. DOI: 10.1161/STR.0000000000000022 24846875

[7] Roth EJ. Heart disease in patients with stroke: incidence, impact, and implications for rehabilitation part 1: Classification and prevalence. Arch Phys Med Rehabil 1993; 74: 752–760. DOI: 10.1016/0003-9993(93)90038-C 8328899

[8] Ekkekakis P, Parfitt G, Petruzzello SJ. The pleasure and displeasure people feel when they exercise at different intensities: decennial update and progress towards a tripartite rationale for exercise intensity prescription. Sports Med 2011; 41: 641–671. DOI: 10.2165/11590680-000000000-00000 21780850

[9] Kaur G, English C, Hillier S. Physiotherapists systematically overestimate the amount of time stroke survivors spend engaged in active therapy rehabilitation: an observational study. J Physiother 2013; 59: 45–51. DOI: 10.1016/S1836-9553(13)70146-2 23419915

[10] Compagnat M, Salle JY, Mandigout S, Lacroix J, Vuillerme N, Daviet JC. Rating of perceived exertion with Borg scale in stroke over two common activities of the daily living. Top Stroke Rehabil 2018; 25: 145–149. DOI: 10.1080/10749357.2017.1399229 29105582

[11] Goikoetxea-Sotelo G, Van Hedel HJA. Defining, quantifying, and reporting intensity, dose, and dosage of neurorehabilitative interventions focusing on motor outcomes. Front Rehabil Sci 2023; 4: 1139251. DOI: 10.3389/fresc.2023.1139251 37637933 PMC10457006

[12] Chen KY, Bassett DR. The technology of accelerometry-based activity monitors: current and future. Med Sci Sports Exerc 2005; 37: S490–S500. DOI: 10.1249/01.mss.0000185571.49104.82 16294112

[13] Joseph C, Strömbäck B, Hagströmer M, Conradsson D. Accelerometry: a feasible method to monitor physical activity during sub-acute rehabilitation of persons with stroke. J Rehabil Med 2018; 50: 429–434. DOI: 10.2340/16501977-2326 29542808

[14] Church G, Smith C, Ali A, Sage K. What is intensity and how can it benefit exercise intervention in people with stroke? A rapid review. Front Rehabil Sci 2021; 2: 722668. DOI: 10.3389/fresc.2021.722668 36188814 PMC9397782

[15] DiPasquale J, Trammell M, Clark K, Fowler H, Callender L, Bennett M, et al. Intensity of usual care physical therapy during inpatient rehabilitation for people with neurologic diagnoses. PM&R 2022; 14: 46–57. DOI: 10.1002/pmrj.12577 33599119

[16] Koopman A, Eken M, Bezeij T, Valent L, Houdijk H. Does clinical rehabilitation impose sufficient cardiorespiratory strain to improve aerobic fitness? J Rehabil Med 2013; 45: 92–98. DOI: 10.2340/16501977-1072 23096222

[17] Barrett M, Snow JC, Kirkland MC, Kelly LP, Gehue M, Downer MB, et al. Excessive sedentary time during in-patient stroke rehabilitation. Top Stroke Rehabil 2018; 25: 366–374. DOI: 10.1080/10749357.2018.1458461 29609499

[18] Lacroix J, Daviet J-C, Borel B, Kammoun B, Salle J-Y, Mandigout S. Physical activity level among stroke patients hospitalized in a rehabilitation unit. PM&R 2016; 8: 97–104. DOI: 10.1016/j.pmrj.2015.06.011 26107540

[19] Folstein MF, Folstein SE, McHugh PR, Fanjiang G. Minimental State Examination: User’s manual. Lutz, FL: Psychological Assessment Resources; 2021.

[20] Manns PJ, Haennel RG. SenseWear armband and stroke: validity of energy expenditure and step count measurement during walking. Stroke Res Treat 2012; 2012: 247165. DOI: 10.1155/2012/247165 23094200 PMC3475303

[21] Mandigout S, Lacroix J, Ferry B, Vuillerme N, Compagnat M, Daviet J-C. Can energy expenditure be accurately assessed using accelerometry-based wearable motion detectors for physical activity monitoring in post-stroke patients in the subacute phase? Eur J Prev Cardiol 2017; 24: 2009–2016. DOI: 10.1177/2047487317738593 29067851

[22] Gilgen-Ammann R, Schweizer T, Wyss T. RR interval signal quality of a heart rate monitor and an ECG Holter at rest and during exercise. Eur J Appl Physiol 2019; 119: 1525–1532. DOI: 10.1007/s00421-019-04142-5 31004219

[23] Demeurisse G, Demol O, Robaye E. Motor evaluation in vascular hemiplegia. Eur Neurol 1980; 19: 382–389. DOI: 10.1159/000115178 7439211

[24] Cheng DK-Y, Dagenais M, Alsbury-Nealy K, Legasto JM, Scodras S, Aravind G, et al. Distance-limited walk tests post-stroke: a systematic review of measurement properties. NRE 2021; 48: 413–439. DOI: 10.3233/NRE-210026 PMC8293643 33967070

[25] MacKay-Lyons M, Billinger SA, Eng JJ, Dromerick A, Giacomantonio N, Hafer-Macko C, et al. Aerobic exercise recommendations to optimize best practices in care after stroke: AEROBICS 2019 update. Phys Ther 2020; 100: 149–156. DOI: 10.1093/ptj/pzz153 31596465 PMC8204880

[26] Gellish RL, Goslin BR, Olson RE, McDonald A, Russi GD, Moudgil VK. Longitudinal modeling of the relationship between age and maximal heart rate. Med Sci Sports Exerc 2007; 39: 822–829. DOI: 10.1097/mss.0b013e31803349c6 17468581

[27] Brawner CA, Ehrman JK, Schairer JR, Cao JJ, Keteyian SJ. Predicting maximum heart rate among patients with coronary heart disease receiving β-adrenergic blockade therapy. Am Heart J 2004; 148: 910–914. DOI: 10.1016/j.ahj.2004.04.035 15523326

[28] Haute Autorité de Santé. Consultation et prescription médicale d’activité physique à des fins de santé chez l’adulte. 2022. [cited 2024 Jun 19]. Available from: https://www.has-sante.fr/upload/docs/application/pdf/2018-10/guide_aps_vf.pdf

[29] Warner A, Vanicek N, Benson A, Myers T, Abt G. Agreement and relationship between measures of absolute and relative intensity during walking: a systematic review with meta-regression. Murias JM, editor. PLoS ONE 2022; 17: e0277031. DOI: 10.1371/journal.pone.0277031 36327341 PMC9632890

[30] Bassett DR. Validity of four motion sensors in measuring moderate intensity physical activity. Med Sci Sports Exerc 2000; 32: S471–S480. DOI: 10.1097/00005768-200009001-00006 10993417

[31] Carbonell-Hernández L, Pastor D, Jiménez-Loaisa A, Ballester-Ferrer JA, Montero-Carretero C, Cervelló E. Lack of correlation between accelerometers and heart-rate monitorization during exercise session in older adults. IJERPH 2020; 17: 5518. DOI: 10.3390/ijerph17155518 32751725 PMC7432703

[32] Kramer S, Johnson L, Bernhardt J, Cumming T. Energy expenditure and cost during walking after stroke: a systematic review. Arch Phys Med Rehabil 2016; 97: 619-632.e1. DOI: 10.1016/j.apmr.2015.11.007 26686877

[33] Compagnat M, Mandigout S, David R, Lacroix J, Daviet JC, Salle JY. Compendium of physical activities strongly underestimates the oxygen cost during activities of daily living in stroke patients. Am J Phys Med Rehabil 2019; 98: 299–302. DOI: 10.1097/PHM.0000000000001077 30358568

[34] Serra MC, Balraj E, DiSanzo BL, Ivey FM, Hafer-Macko CE, Treuth MS, et al. Validating accelerometry as a measure of physical activity and energy expenditure in chronic stroke. Top Stroke Rehabil 2017; 24: 18–23. DOI: 10.1080/10749357.2016.1183866 27322733 PMC5125839

[35] Cosoli G, Spinsante S, Scalise L. Wrist-worn and chest-strap wearable devices: systematic review on accuracy and metrological characteristics. Measurement 2020; 159: 107789. DOI: 10.1016/j.measurement.2020.107789

[36] Mühlen JM, Stang J, Lykke Skovgaard E, Judice PB, Molina-Garcia P, Johnston W, et al. Recommendations for determining the validity of consumer wearable heart rate devices: expert statement and checklist of the INTERLIVE Network. Br J Sports Med 2021; 55: 767–779. DOI: 10.1136/bjsports-2020-103148 33397674 PMC8273688

[37] Pollock ML, Foster C, Rod JL, Wible G. Comparison of methods for determining exercise training intensity for cardiac patients and healthy adults. In: Kellermann JJ, editor. Advances in cardiology [serial on the Internet]. S. Karger AG; 1982, p. 129–133. DOI: 10.1159/000407132 6983817

[38] Dos Reis MTF, Aguiar LT, Peniche PDC, Faria CDCDM. Are age-predicted equations valid in predicting maximum heart rate in individuals after stroke? Disabil Rehabil 2023; Aug 22 [online ahead of print]. DOI: 10.1080/09638288.2023.2247981 37606274

[39] Brubaker PH, Kitzman DW. Chronotropic incompetence: causes, consequences, and management. Circulation 2011; 123: 1010–1020. DOI: 10.1161/CIRCULATIONAHA.110.940577 21382903 PMC3065291

[40] Gonçalves C, Raimundo A, Abreu A, Bravo J. Exercise Intensity in patients with cardiovascular diseases: systematic review with meta-analysis. IJERPH 2021; 18: 3574. DOI: 10.3390/ijerph18073574 33808248 PMC8037098

